# Strategies for Efficient Targeting of Tumor Collagen for Cancer Therapy

**DOI:** 10.3390/cancers14194706

**Published:** 2022-09-27

**Authors:** Silvia Baldari, Francesca Di Modugno, Paola Nisticò, Gabriele Toietta

**Affiliations:** Tumor Immunology and Immunotherapy Unit, IRCCS Regina Elena National Cancer Institute, Via Chianesi 53, 00144 Rome, Italy

**Keywords:** collagen, tumor microenvironment, extracellular matrix, cancer-associated fibroblasts, cancer therapy, stromal cells, tumor stroma, cancer immunotherapy, fibrillar collagen, tumor heterogeneity

## Abstract

**Simple Summary:**

The tumor microenvironment encompasses the cellular and extracellular matrix components that support and shape the three-dimensional framework in which solid tumors develop and grow. The extracellular matrix of the tumor is characterized by increased deposition and aberrant architecture of collagen fibers. Therefore, as a key mechanical component of the tumor microenvironment, collagen plays a critical role in cancer progression, metastasis, and therapeutic response. To boost the efficacy of current anticancer therapies, including immunotherapy, innovative approaches should take into account strategies directed against the dysregulated non-cancer cell stromal components. In the current review, we provide an overview of the principal approaches to target tumor collagen to provide therapeutic benefits.

**Abstract:**

The tumor stroma, which comprises stromal cells and non-cellular elements, is a critical component of the tumor microenvironment (TME). The dynamic interactions between the tumor cells and the stroma may promote tumor progression and metastasis and dictate resistance to established cancer therapies. Therefore, novel antitumor approaches should combine anticancer and anti-stroma strategies targeting dysregulated tumor extracellular matrix (ECM). ECM remodeling is a hallmark of solid tumors, leading to extensive biochemical and biomechanical changes, affecting cell signaling and tumor tissue three-dimensional architecture. Increased deposition of fibrillar collagen is the most distinctive alteration of the tumor ECM. Consequently, several anticancer therapeutic strategies have been developed to reduce excessive tumor collagen deposition. Herein, we provide an overview of the current advances and challenges of the main approaches aiming at tumor collagen normalization, which include targeted anticancer drug delivery, promotion of degradation, modulation of structure and biosynthesis of collagen, and targeting cancer-associated fibroblasts, which are the major extracellular matrix producers.

## 1. Introduction

Tumor and tumor-associated stromal cells promote the production and remodeling of the extracellular matrix (ECM) to create a tumor microenvironment (TME) that supports cancer growth, metastatic dissemination, and immune evasion and affects the patient’s response to therapy [1]. ECM is composed of a vast array of proteins, proteoglycans and glycosaminoglycans organized in a complex and dynamic three-dimensional network. The members of the collagen family are the most abundant (up to 90%) proteins in the ECM [2]. Collagen synthesis and assembly are a complex, multistep process involving different specific enzymes and molecular chaperones that are tightly regulated to preserve tissue homeostasis. Collagens are composed of three homo- or hetero-trimeric polypeptide chains (α chains), which are synthesized as pre-pro-collagens that undergo several post-translational modifications, including proline and lysine hydroxylation and glycosylation, in the endoplasmic reticulum. Three post-translationally modified pro-α chains form a procollagen molecule, which, upon secretion into the extracellular space, is proteolytically cleaved. Triple-helical procollagen is transported across the Golgi complex, self-assembled into collagen fibrils and exported into the ECM. The fibrils are then stabilized by the formation of covalent crosslinks and aggregation of multiple collagen fibrils to finally produce collagen fibers [3]. Among the 28 isoforms of collagen identified in humans, the types I, II, III, V, XI, XXIV and XXVII constitute the sub-group of fibrillar collagen, which organizes a three-dimensional framework that supports the ECM’s mechanical strength and regulates cell adhesion, migration, differentiation, and survival [4]. Each collagen isoform has a distinct tissue distribution (Supplemental Table S1) and might exert diverse functions in cancer-associated processes [5]. In particular, fibrillar collagen types I and III are the most abundant isoforms of collagen and have been associated with different types of tumors, including bone, breast, colorectal, ovarian, lung, head and neck, and pancreatic cancers [6].

In cancer, the ECM structure, physical properties, metabolism, and function are highly dysregulated. In particular, the tumor ECM is more abundant, condensed, and stiffer than the ECM in the surrounding healthy tissue, leading to increased interstitial fluid pressure and making the tumor less accessible to nutrients, oxygen, immune cells, and therapeutic drugs [7]. Above all, collagens are upregulated in several types of cancer, such as oral squamous cell carcinoma, breast, pancreatic, and gastric cancers; moreover, high collagen expression correlates with poor overall survival and affects the response to chemo-, radio- and immuno-therapies [8,9]. Collagen has a prognostic and predictive value in different types of solid tumors, including breast, prostate, lung, liver, colon, and pancreatic cancers [10,11]. In particular, oriented collagen around tumor cells [12] and the identification of distinct collagen organization patterns, termed tumor-associated collagen signatures (TACS), are indicators of disease progression [13]. Recently, the predictive value of collagen has been extended from tissue to blood, as non-invasive determination of serum collagen fragments has been proposed for the optimization of patient selection to improve the efficacy of immune checkpoint inhibitor (ICI) immunotherapy [14].

Cancer cell behavior is modulated via a biochemical and biomechanical cross-talk with stromal cells, mainly cancer-associated fibroblasts (CAFs) [15,16]. Different subsets of CAFs have been identified on the basis of their gene expression, phenotypic marker profiles and functions [17,18]. Among these sub-types, the myofibroblast-like CAFs (myCAFs) express high levels of fibroblast activation protein (FAP), secrete cytokines, chemokines, and extracellular vesicles and produce a dense collagen-rich ECM that modulates the infiltration of immune cells within the TME, suppressing antitumor immunity [18,19,20,21].

**Figure 1:**
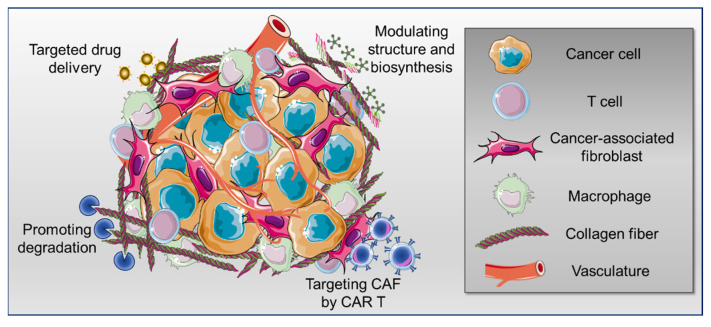
Schematic representation of different strategies of therapeutic targeting of tumor collagen. The figure was partly generated by adapting Servier Medical Art pictures provided by Servier, licensed under a Creative Commons Attribution 3.0 unported license.

In different types of solid tumors, poor prognosis and resistance to immunotherapy have been associated with the increased density, degree of alignment and crosslinking of fibrillar collagens, mainly type I [22,23]. Interestingly, a critical role of collagen type XII in the regulation of collagen type I organization has been recently elucidated in breast cancer [24]. Altogether, a balanced degree of collagen production and degradation is essential for an optimal immunotherapy response [14]. Therefore, recently, anticancer strategies aiming at interfering with collagen-immune cell interactions have been considered [25,26]. Herein, we provide an overview of the current advances and challenges of the main approaches targeting collagen for tumor ECM normalization that might lead to synergistic therapeutic efficacy when used in combination with other strategies, including immunotherapy (Figure 1).

## 2. Collagen Targeting for Anticancer Drug Delivery

The interactions of different proteins with several types of collagen are mediated by specific collagen-binding domains (CBD) [27]. Engineering antibodies, drugs, or cytokines with a CBD allows for the targeting and release of the CBD-associated biomolecules into the tumor collagen scaffold, reducing off-target effects, decreasing toxicity upon systemic administration, and increasing localized retention, thereby enhancing their therapeutic efficacy [28]. As an example, Liang et al. demonstrated that the fusion of a recombinant protein containing the EGFR binding fragment of cetuximab with a CBD results in specific targeting and improved penetration into squamous carcinoma A431 cell xenografts [29]. A similar strategy was used to obtain CBD conjugation to immune checkpoint inhibitor antibodies and fusion to interleukin-2 (IL-2) [30]. Interestingly, in different tumor models, both CBD-fused IL2 and CBD-conjugated checkpoint inhibitors showed enhanced antitumor efficacy and reduced associated toxicity compared with their unmodified counterparts. Moreover, CBD fusion to IL-12 has been described as resulting in systemic toxicity reduction and synergy with immune checkpoint inhibitor therapy [31]. Improvements in the efficacy of cytokine therapy for cancer treatment were also achieved by fusing IL-2 and IL-12 to the collagen-binding protein lumican to potentiate cytokine specificity and local retention and reduce systemic toxicity [32]. Among the strategies to ameliorate in situ drug delivery, the combination of collagen-derived hydrogels and CBD has also been investigated; for instance, localized and controlled delivery of immunotherapeutics has been achieved by the implantation of a collagen hydrogel loaded with interferon-alpha 2b fused to a collagen-binding domain [33]. The above-mentioned studies collectively demonstrate the possibility of targeting both cytokines and immune checkpoint inhibitors by engineering them with collagen-binding peptides or proteins to achieve improved immunotherapy safety and efficacy.

Some recent investigations showed that albumin, the most abundant plasma protein, can also be used as a carrier to improve the pharmacokinetics, solubility and serum stability of anticancer drugs [34]. Moreover, albumin accumulates in the TME since it is used as an energy source by fast-growing cancer cells [35]. Exploiting these favorable pharmacological features, Sasaki et al. developed a strategy to obtain collagen-binding serum albumin drug conjugates. In particular, doxorubicin was conjugated with albumin fused with a CBD and used to treat a murine model of colon carcinoma. In combination with an anti–PD-1 checkpoint inhibitor, the treatment resulted in complete tumor regression by virtue of the significantly higher doxorubicin accumulation observed within the TME [36]. Chemotherapeutic drug delivery by anti-collagen 4 immunoconjugates has also been described as a strategy for tumor stroma targeting [37].

Other than maximizing the therapeutic efficacy of anticancer drugs, tumor collagen drug-targeting has also been considered for enhancing the pharmacokinetics of diagnostic compounds [28]. For instance, the development of a collagen-targeted MRI contrast agent has been described to achieve high sensitivity at low dosage, reduce metal toxicity, and facilitate disease progression monitoring and the early detection of liver metastasis [38,39].

## 3. Strategies to Promote Tumor Collagen Degradation

### 3.1. Collagenase Treatment

The administration of different matrix-modulating enzymes, including collagenase, relaxin and hyaluronidase, has been used to promote the degradation of the extracellular matrix (ECM) components aiming at tumor stiffness reduction [40]. In particular, studies performed on animal models indicate that collagenase treatment can improve the diffusion and the uptake of therapeutic macromolecules, nanoparticles, and gene therapy vectors into solid tumors by approximately 2-fold on average [41,42,43]. The clinical significance of this relatively modest effect is controversial, and it is likely dependent on the type and on the stage of the tumor, the delivery route and the duration of the treatment [40,42]. Furthermore, the products of collagen degradation can still promote cancer angiogenesis and metastasis [44]. Therefore, toxicity and immunogenicity of administered collagenase, off-target effects on non-tumor tissues and the possible increase in the tumor metastasis potential need to be precisely addressed before clinical translation.

### 3.2. Collagenase Encapsulated Nanoparticles and Hydrogels

Advances in nanotechnology and the engineering of hydrogel materials have provided new opportunities for controlled local delivery of ECM-degrading enzymes. Collagenase functionalization of nanoparticles has been shown to promote the degradation of extracellular stroma in different tumor experimental models, thereby enhancing the permeability and retention of antitumor drugs [45,46,47,48]. Pan et al. described a localized co-delivery strategy into HER2-positive BT474 tumor-bearing mice of collagenase and trastuzumab by using a thermosensitive hydrogel, suggesting that this delivery route may promote the penetration of the therapeutic antibody into deeper tumor tissues [49].

### 3.3. Protein-Free Collagen Degradation

As a strategy to promote the degradation of tumor collagen without the use of collagen-degrading enzymes, Dong et al. described the use of nanoparticles loaded with a chemotherapeutic agent, doxorubicin, and a nitric oxide (NO) donor. The loaded NO induced the activation of resident matrix metalloproteinases (MMPs) that degrade the collagen in the TME, further facilitating the penetration of the nanoparticles and their therapeutic payload in the orthotopic 4T1 breast cancer model [50]. Differently from the use of collagen-degrading enzymes, this alternative strategy leads to increased tumor penetration of both the loaded cargo and the nanoparticle, thus leading to improved anticancer efficacy with reduced toxicity.

### 3.4. Collagen-Degrading Bacteria

Motile bacteria are promising anticancer drug delivery vectors by virtue of their proteolytic activity toward ECM components that promote solid tumor colonization [51,52]. Recently, the engineering of bacteria to promote ECM degradation has been proposed as an innovative strategy to modify the immune landscape of the TME. In particular, engineered collagen I-degrading *Salmonella typhimurium* effectively targets collagen within the pancreatic ductal adenocarcinoma (PDAC) tissue, reduces the frequency of suppressive intratumoral cells and improves the efficacy of combined immunotherapy treatments [53].

### 3.5. Degradation of Tumor Extracellular Matrix Mediated by Armed Oncolytic Virus

Oncolytic viruses (OVs) can specifically replicate in tumor cells inducing their lysis; moreover, OVs may also target tumor stromal cells, including cancer-associated fibroblasts (CAFs), leading to profound alterations within the TME [54,55]. As OV engineering allows for the expression of transgenes that may enhance the antitumor immune responses and the TME remodeling, oncolytic adenoviral viruses expressing relaxin [56,57], decorin [58,59,60,61] and MMP-8 [62] have been generated to decrease the synthesis or promote the degradation of components of the ECM, including collagen fibers, thus supporting the viral spread and, consequently, improving virotherapy therapeutic efficacy [63]. Recently, Zhang et al. observed a synergistic antitumor effect of the combination between an oncolytic adenovirus carrying decorin with a CAR T cell therapy targeting carbonic anhydrase IX. In particular, in a xenograft model of human renal carcinoma, this combined therapy altered the distribution of collagen fibers within the TME, promoting the efficacy of CAR T cells by enhancing T cell persistence [64]. Oncolytic viruses have also been engineered to produce bispecific T cell engagers (BiTEs) to target some tumor stromal components to promote antitumor effects [65,66,67].

## 4. Strategies to Modulate Collagen Structure and Biosynthesis

### 4.1. Modulation of Lysyl Oxidase Enzymatic Activity

Lysyl oxidase (LOX) and its family members LOX-Like 1-4 (LOXL1-4) are amine oxidases that play a role in ECM remodeling and in the crosslinking of collagens and elastin by catalyzing the deamination of lysine and hydroxylysine residues. Collagen crosslinking status is a major determinant of tissue stiffness [68]. As a consequence, aberrant expression of lysyl oxidases is implicated in cancer progression, metastasis and development of tumor chemoresistance [69]. For this reason, the LOXL family has been considered an attractive drug target for anticancer therapy to achieve the reduction of ECM stiffness [70,71]. Different approaches have been developed for lowering LOXL activity, including the following: (i) beta-aminopropionitrile (BAPN), which acts as a potent irreversible inhibitor of LOX activity [72]; accordingly, BAPN-induced modulation of tumor stiffness increased intratumoral T cell migration and infiltration [73]; (ii) simtuzumab, an anti-LOXL2 antibody, which inhibits LOXL2 activity in preclinical models, but the observed clinical benefits were limited [74]; (iii) bone morphogenetic protein 1 (BMP1), which cleaves and activates LOX precursor, thus, inhibition of BMP1 has been shown to reduce the levels of active LOX [72]; (iv) LOXL family members, which are copper-binding enzymes. Since the copper-binding domain is important for the catalytic activity, copper chelation therapy might result in the indirect inhibition of LOX and LOXL functions and promote TME remodeling [75]. A recent phase II clinical trial using tetrathiomolybdate in high-risk triple-negative breast cancer patients has indeed shown that copper chelation decreases collagen crosslinking and may increase T-cell infiltration into breast pre-metastatic sites [76].

### 4.2. Modulation of Collagen Glycation-Related Crosslinking

Advanced glycation end products (AGEs) are heterogeneous compounds formed by non-enzymatical glycation reactions between reducing sugars and the amino groups of proteins, lipids, and nucleic acids. AGEs can be produced endogenously during physiological metabolism, can be formed in excess in patients with diabetes, or are assumed through foods processed at high temperatures and by tobacco smoking. AGEs interact with the receptor for advanced glycation end products (RAGE), leading to inflammatory, angiogenic, and fibrotic reactions that promote tumorigenesis and the progression of other pathological conditions [77,78]. Interestingly, Krisanits et al. have recently observed that in mice fed with a high-AGE diet, pro-tumorigenic effects on prostate cancer depend on RAGE expression and activation in CAFs [79]. This opens the perspective of the targeted inhibition of RAGE or its ligands for treating solid cancers [80]. Importantly, AGEs may also promote cancer progression and metastasis in a RAGE-independent manner as AGEs produce collagen intermolecular crosslinks by bonding the free amino groups of neighboring molecules. AGE-mediated crosslinking alters collagen tertiary structure, charge and physiological function, decreasing its proteolytic degradation and increasing stiffness [81]. Thus, inhibitors of the absorption of exogenous AGEs, anti-glycation agents, and compounds that induce breakage and reversal of AGEs have been considered for cancer therapy [82].

### 4.3. Collagen Biosynthesis Inhibition by Antifibrotic Drugs

The repurposing of approved antifibrotic medicines, which target collagen synthesis and maturation, as anticancer drugs has attracted increasing interest [40]. For instance, halofuginone, an antifibrotic agent that blocks fibroblasts’ Smad3 activation upon transforming growth factor beta (TGF-β1) stimulation [83], is able to affect collagen matrix architecture, leading to improved drug delivery and immune infiltration into a PDAC model [84]. Similarly, pirfenidone, another antifibrotic agent used for treating idiopathic pulmonary fibrosis, inhibits collagen production in breast cancer-associated fibroblasts [85], fibroblast activation and tumor-stroma interaction in non-small cell lung cancer [86,87]. Among the other antifibrotic drugs, losartan is a Food and Drug Administration-approved anti-hypertensive drug that also exerts antifibrotic effects by suppressing the levels of active TGF-β1. The use of losartan at doses having a minimal effect on arterial blood pressure reduced collagen I levels in different murine cancer models and improved the penetration of nanotherapeutics into the tumor [88,89]. Interestingly, tamoxifen, a selective estrogen receptor modulator, has been shown to reduce the levels of hypoxia-inducible factor-1 alpha (HIF-1α) and, consequently, decrease collagen deposition, alter fiber alignment and reduce tissue stiffness in a murine model of PDAC [90]. Therefore, the use of antifibrotic drugs may promote tumor tissue remodeling and support increased drug delivery; nonetheless, the effects on the tumor spread of long-term antifibrotic therapy may represent a critical issue [40,91].

### 4.4. Modulation of Proline Incorporation and Hydroxylation

Proline residues, which account for approximately 10% of the total amino acids, play an essential role in the synthesis and structure of collagen. The incorporation of proline analogs in the procollagen polypeptide chains can lead to the disruption of the conformation of the collagen triple-helix and promote collagen degradation [92]. Accordingly, supplementation of the proline analog thiaproline in the drinking water reduced collagen biosynthesis and malignant mesothelioma growth in a murine model [93]. Furthermore, post-translational modification of the (2S)-proline residues catalyzed by the collagen prolyl 4-hydroxylases (CP4Hs) is required for the collagen triple helix stability. Inhibitors of CP4Hs have been developed as potential anticancer agents [94], for instance, in breast cancer [95] and colorectal [96] mouse models, but heavy toxicity and off-target effects currently limit their clinical translation [97].

## 5. Strategies for Targeting Cancer-Associated Fibroblasts as the Major Extracellular Matrix Producers

Cancer-associated fibroblasts (CAFs) comprise a highly heterogeneous group of stromal cells that are the major producers of collagen and ECM crosslinking enzymes within the TME [21,98]. Collagen deposition during tumor progression leads to increased tissue stiffness and desmoplasia [6,99], thus affecting intratumoral immune cell migration [22]. Additionally, immunosuppressive CAF secretome contributes to the inhibition of T cell function within the TME. The mechanisms by which some sub-populations of CAFs support tumorigenesis are conserved in different types of cancer; thus, anticancer therapies targeting CAFs can potentially be effective against a broad spectrum of solid tumors. Moreover, albeit highly heterogeneous, CAFs are more genetically stable than cancer cells and consequently less prone to antigen escape and acquired drug resistance. Therefore, different strategies aiming at enhancing antitumor immunity by pro-tumorigenic CAF depletion or by inhibition of their activation and function have been explored [100].

One of the most attractive strategies to achieve CAF depletion is by targeting CAFs by CAR T cell therapy. Different sub-populations of CAFs may exert pro- or antitumorigenic effects; therefore, an accurate target selection is needed to identify the fibroblast sub-population(s) to be depleted. Fibroblast activation protein (FAP) is a membrane protease preferentially expressed by activated cardiac fibroblasts [101] and by pro-tumorigenic CAFs; thus it has been considered a suitable target for CAR T cell therapy directed against immunosuppressive TME components [102]. This hypothesis has been confirmed by several preclinical studies performed using anti-FAP CAR T cell-mediated therapy in different murine models of lung cancer [103,104,105,106], providing support for subsequent clinical translation [104,107,108]. In particular, the recently published preliminary data of a phase I clinical trial for pleural mesothelioma using CAR T cells targeting FAP indicate that the procedure is feasible [109] and not associated with on-target off-tumor toxicity, otherwise observed in an earlier preclinical study [110]. Some matrix metallo- and cysteine-proteases can cleave collagen, generating unstable fragments which are internalized for degradation by two endocytic collagen receptors, the mannose receptor (MRC1/CD206) and the urokinase plasminogen activator receptor-associated protein (uPARAP/Endo180/CD280/MRC2) [111]. Interestingly, MRC2 expression identifies a sub-population of matrix-remodeling CAFs [112,113], opening the consideration of MRC2 as a putative stromal target for CAR T therapy in solid tumors. Additional strategies of CAF-targeted therapy aiming at interfering with their role in TME remodeling include the possibility of reprogramming CAF to a non-tumorigenic phenotype or the blocking of CAF-induced pro-tumorigenic molecular signaling on target cells [114].

The growing knowledge of the dynamic nature of the TME and on the molecular and functional heterogeneity of different CAF sub-types will support the development of innovative cancer therapeutic strategies targeting the tumor stromal compartment [115].

## 6. Collagen and Immune Cell Infiltration: A Link to the Efficacy of Immunotherapies

ICI immunotherapy is a breakthrough in cancer therapy; however, a fraction of patients does not respond, due to innate or acquired resistance, raising the need to more precisely unveil the mechanisms of resistance [116]. ECM remodeling, which occurs in solid tumors with increased stiffness and high-collagen content, influences immune cell behavior, thereby affecting the response to immunotherapy. In this scenario, the emergent research field of matrix immunology is rapidly evolving [23]. In particular, different studies have explored the mechanisms underlying the relationship between a dense ECM, mainly determined by fibronectin and the collagen fibers’ density, alignment and organization, and inhibition of T cell migration into the tumor core [117,118,119,120].

The collagen receptor discoidin domain receptor 1 (DDR1) induces immune exclusion by promoting collagen fiber alignment; therefore, treatment with a DDR1-neutralizing antibody that disrupts collagen fiber alignment increases immune infiltration and inhibits tumor growth in triple-negative breast cancer [121]. As mentioned above, the antifibrotic agent halofuginone induces greater cytotoxic T cell immune infiltrate, intratumoral necrosis and reduced tumor volume in a genetically-engineered mouse model of PDAC [84]. The role of collagen deposition as an inhibitor of immune cell infiltration and activation has recently been related to the high expression of the transcription factor sine oculis homeobox 1 (SIX1). In particular, the requirement of SIX1 for the expression of multiple collagen genes occurs via the TGF-β signaling pathway by the upregulation of the transforming growth factor beta receptor 2 expression [122]. ECM-associated signaling pathways and collagen density have been reported to be associated with increased regulatory T cell (Treg) infiltration in triple-negative breast cancer [123]. Recently, the research group of Raghu Kalluri demonstrated that an abnormal homotrimer variant of the type I collagen contributes to PDAC initiation and progression; the deletion of this variant in cancer cells promotes T cell infiltration and renders the tumor responsive to ICI immunotherapy [124].

Strategies to target the ECM, in particular collagens, have been proposed in association with immune checkpoint inhibitors and resulted in increased efficacy, at least in murine models. The inhibition of LOX enzymatic activity, in combination with anti-PD-1 administration, improved the effector CD8^+^ T cell infiltration and accumulation in tumors and increased the efficacy of PD-1 blockade in a murine model of PDAC [73]. Chen et al. recently demonstrated that the disruption of a collagen deposition program activated by IL-17 signaling, which drives immune exclusion, increases anti–PD-L1–mediated tumor regression in a murine model of cutaneous squamous cell carcinoma [125]. Deletion of the MRC2 collagen receptor, which is highly expressed in the immunosuppressive ECM-myCAF sub-type, facilitates CD8^+^ T cell infiltration and enhances sensitivity to ICI in a murine model of mammary tumor [112]. Accordingly, MRC2 high expression in human clinical samples is associated with poor responses to αPD-1 therapy [113].

The immunomodulatory function of collagens also relies on their property to act as a ligand for immune inhibitory receptors such as the leukocyte-associated immunoglobulin-like receptor-1 (LAIR-1). Collagen-induced T cell exhaustion occurs through the LAIR-1 receptor, and its signaling abrogation in a mouse model of lung adenocarcinoma sensitizes resistant tumors to PD-1 blockade [8]. Collagen production is dependent on TGF-β signaling and, in the last few years, the effectiveness of the combination of inhibitory checkpoint blockade and TGF-β signaling targeting has been largely described [126]. Horn and colleagues evaluated the effect of co-inhibition of TGF-β, PD-L1, and LAIR-1 signaling in murine tumor models of mammary carcinoma and colon cancer. This strategy decreased ECM denatured collagen content, enhanced the infiltration of activated CD8^+^ T cells and reduced tumor growth [127]. Furthermore, since collagen I deposition depends on the activation of the focal adhesion kinase (FAK), FAK inhibition has been shown to reduce fibrosis and decrease collagen deposition; notably, FAK inhibition also rendered PDAC mice responsive to chemo- and immunotherapy [128].

Altogether, these data indicate that tumor collagen exerts relevant immunomodulatory functions that need to be considered to further elucidate the mechanisms of resistance, improve the stratification of patients and design effective strategies for combined immunotherapy.

## 7. Conclusions

As a key player in TME structure and mechanotransduction, collagen plays a critical role in cancer progression, metastasis, and therapeutic response, and it may, therefore, represent a possible target for tumor therapy. In particular, combined strategies targeting cancer cells as well as stromal cells can improve cancer therapy efficacy. To this aim, cancer-collagen-targeting of drugs and immunoconjugates, as well as strategies aiming at promoting tumor collagen degradation or reducing its biosynthesis, have been developed. On the other hand, studies on a murine model of pancreatic cancer have shown that collagen I deletion in myofibroblasts augments immune suppression and accelerates tumor progression, thus highlighting the complexity of the stromal contributions to tumor progression [129]. As a matter of fact, collagens may exert both pro- and anti-neoplastic effects in a context-dependent manner, as recently reviewed elsewhere [5,25]; moreover, different CAF sub-populations within the TME have opposing functions on pancreatic ductal adenocarcinoma progression and therapy response [130]. Indeed, the spatial heterogeneity and temporal dynamics of the TME composition in different types of solid tumors are the major challenges to the effective selective targeting of the tumor stroma. For this reason, a better definition and characterization of the stromal components that support collagen pro-tumorigenic production and crosslinking and are involved in an immune-excluded phenotype are essential for developing future therapeutic treatments.

## Supplementary Materials

The following supporting information can be downloaded at: https://www.mdpi.com/article/10.3390/cancers14194706/s1, Table S1: Collagen types, sub-groups and their main tissue distribution. Reference [131] is citied in the Supplementary Materials.
