# Supplementary material for: Strategies for Efficient Targeting of Tumor Collagen for Cancer Therapy
Source: Cancers (Basel). 2022 Sep 27;14(19):4706. doi: 10.3390/cancers14194706 (PMC9563908; doi:10.3390/cancers14194706)
Supplement: Supplementary file 1 [file cancers-14-04706-s001.zip › cancers-1914315-supplementary.pdf]

cancers

MDPI

Supplementary Materials: Strategies for Efficient Targeting of Tumor Collagen for Cancer Therapy

Silvia Baldari, Francesca Di Modugno, Paola Nisticò and Gabriele Toietta

Table S1. Collagen types, sub-groups and their main tissue distribution.

Collagen Type	Collagen Sub-Group	Tissue Distribution in Healthy Tissue	Tissue Distribution in Associated Cancers
I	Fibrillar	Connective tissues, skin, dermis, cornea, tendon, bone	Bone, breast, colon, ovary, lung, pancreas
II	Fibrillar	Cartilage	Bone (chondrosarcoma)
III	Fibrillar	Dermis, aorta and blood vessels, uterus, intestine and reticular connective tissue of visceral organs and adipose tissue	Breast, colon, head and neck, pancreas
IV	Network-forming	Basal laminae	
V	Fibrillar	Cornea, placental/embryonic tissue	Breast
VI	Beaded filaments	Ubiquitous, but particularly in skeletal muscle	Breast, colon, ovary, brain (glioma), skin (melanoma), pancreas
VII	Anchoring fibrils	Skin, amniotic membrane, cornea, mucosal epithelium	
VIII	Network-forming	Descemet's membrane of the eye, endothelial cells	
IX	FACIT	Cartilage	
X	Network-forming	Hypertrophic cartilage	
XI	Fibrillar	Cartilage, intervertebral disc, skeletal muscle, lung	Head and neck, breast, stomach, lung, ovary, pancreas
XII	FACIT	Cartilage, bone, dense connective tissue	Breast
XIII	Transmembrane	Cutaneous tissue; neuromuscular junctions	
XIV	FACIT	Dermis, tendon, cartilage	Head and neck
XV	Multiplexin	Placenta, kidney, heart, ovary, testis	
XVI	FACIT	Cartilage, heart, kidney, muscle	
XVII	Transmembrane	Skin hemidesmosomes	
XVIII	Multiplexin	Kidney, placenta, lung, liver, skin	
XIX	FACIT	Interneurons	
XX	FACIT	Corneal epithelium, skin, cartilage, and tendon	
XXI	FACIT	Blood vessel walls	
XXII	FACIT	Tissue junctions	
XXIII	Transmembrane	Cutaneous tissue	Prostate
XXIV	Fibrillar	Differentiating osteoblasts, developing cornea	
XXV	Transmembrane	Neurons, hippocampus	
XXVI	FACIT	Testis and ovary	
XXVII	Fibrillar collagen	Cartilage, embryonic eye, coronary arteries, dermis	
XXVIII	Network-forming	Non-myelinated regions of the peripheral nervous system	

FACIT: fibrillar-associated collagens with interrupted triple-helices. Adapted with modifications from [1–3].

References

1. Nissen, N.I.; Karsdal, M.; Willumsen, N. Collagens and cancer associated fibroblasts in the reactive stroma and its relation to Cancer biology. *J. Exp. Clin. Cancer Res.* **2019**, *38*, 115. https://doi.org/10.1186/s13046-019-1110-6.
2. Fuller, A.M.; Eisinger-Mathason, T.S.K. Context matters: Response heterogeneity to collagen-targeting approaches in desmoplastic cancers. *Cancers* **2022**, *14*, 3132. https://doi.org/10.3390/cancers14133132.
3. Sorushanova, A.; Delgado, L.M.; Wu, Z.; Shologu, N.; Kshirsagar, A.; Raghunath, R.; Mullen, A.M.; Bayon, Y.; Pandit, A.; Raghunath, M.; et al. The collagen suprafamily: From biosynthesis to advanced biomaterial development. *Adv. Mater.* **2019**, *31*, e1801651. https://doi.org/10.1002/adma.201801651.
